# Cross resistance emergence to polymyxins in *Acinetobacter baumannii* exposed in vitro to an antimicrobial peptide

**DOI:** 10.1038/s44259-025-00120-4

**Published:** 2025-05-29

**Authors:** Emily Ritz, Tiffany Rossel, Nicolas Jacquier

**Affiliations:** Institute of Microbiology, University Hospital Centre and University of Lausanne, Lausanne, Switzerland

**Keywords:** Microbiology, Antimicrobials, Bacteria, Pathogens

## Abstract

Multidrug-resistant bacteria are a growing public health concern. Antimicrobial peptides (AMPs) are proposed alternatives to classical antibiotics towards infections caused by resistant bacteria. TAT-RasGAP_317-326_ is an AMP able to target Gram-negative bacteria and is especially efficient towards *Acinetobacter baumannii*. In this study, we performed in vitro resistance selection on several *A. baumannii* strains, in order to determine to which extent these bacteria can develop resistance to TAT-RasGAP_317-326_. *A. baumannii* rapidly developed resistance to TAT-RasGAP_317-326_ and subsequently, in approximately half of the cases, cross-resistance to last-resort polypeptidic antibiotics polymyxins. Cross-resistant isolates predominantly bore mutations in the *pmrAB* operon, involved in modulation of lipopolysaccharides' charge at the bacterial surface, similarly to polymyxin-resistant clinical isolates. We thus show here that contact of *A. baumannii* with an AMP structurally different from polymyxins can induce unexpected cross-resistance towards them. This indicates that precautions must be taken for the clinical application of AMPs.

## Introduction

Antibiotic resistance is a rising concern for modern medicine, making it crucial to develop alternative treatment strategies. In this context, antimicrobial peptides (AMPs) have been proposed as such an alternative. AMPs are naturally occurring peptides produced by a wide variety of organisms as part of their first line of defense against pathogens^1^. They possess remarkably diverse sequences and sizes and can be produced either by ribosomes or via enzymatic complexes. These molecules can contain non-classical amino acids and be highly modified and even cyclized^2^. Only a limited number of AMPs have been approved for clinical use^3^. This is the case for polymyxins (colistin and polymyxin B), which are used as last-resort antimicrobial agents, despite potentially important side effects^4^. AMPs are proposed as promising antimicrobial agents since it is usually believed that the development of resistance towards them is very limited. This has been challenged by studies showing that bacteria can develop resistance to AMPs^5,6^. Resistance to AMPs seems generally specific to a subclass of AMPs, and only limited cross-resistance could be observed^7,8^. However, it was recently shown that resistance to colistin detected in clinical strains can cause moderate cross-resistance to host AMPs, inducing an increased virulence of the resistant strains^9^. Nevertheless, multidrug-resistant (MDR) bacteria are usually sensitive to AMPs^10^, indicating that broad cross-resistance to both classical antibiotics and AMPs is unlikely. Cross-resistance development was mainly studied in the model organism *Escherichia coli*^8^. Whether these observations can be generalized to other pathogenic bacteria is still an open question.

*Acinetobacter baumannii* is a Gram-negative opportunistic human pathogen. It is a common agent of nosocomial infections that colonizes both the respiratory and urinary tracts. A strong advantage of *A. baumannii* is its capacity to form biofilms on biotic and abiotic surfaces^11^. These biofilms can be highly tolerant to antimicrobial agents and disinfectants. *A. baumannii* shows high genome plasticity that allows this bacterium to rapidly gain resistance to antibiotics such as carbapenem^12^. For this reason, carbapenem-resistant *A. baumannii* was highlighted by the WHO as a critical priority pathogen for research and development of new antibiotics. Polymyxins are last-resort antibiotics against these carbapenem-resistant *A. baumannii*. Nevertheless, the emergence of polymyxin-resistant strains of *A. baumannii* has also been documented^13^. Resistance to polymyxins usually involves mutations in genes encoding the two-component system PmrAB, which induce modifications of the lipopolysaccharide (LPS), also known as lipooligosaccharide (LOS) in *A. baumannii*, via the addition of positive charges to Lipid A^14^. AMPs have been pointed as promising antimicrobial agents towards *A. baumannii*^15^. However, little is known about the potential of *A. baumannii* to acquire resistance towards these AMPs.

TAT-RasGAP_317-326_ was first developed as an anticancer peptide and was later characterized as an antibacterial agent^16^. This cationic peptide contains uniquely proteinogenic amino acids and was produced as an all D- retro-inverted peptide. It showed promising activity towards Gram-negative bacteria, especially towards both *A. baumannii* ATCC 19606 strain (MIC of 8 µg/ml) and MDR clinical isolates (MIC of 16 µg/ml)^16^. This peptide also had inhibitory activity against an in vitro model of *A. baumannii* biofilm, with a minimal biofilm inhibition concentration between 32 and 64 µg/ml when used alone, and 4 µg/ml when used in combination with 4 µg/ml of meropenem^17,18^. It also partially affects the viability of mature biofilm alone or in combination with meropenem^17,18^. In addition, the efficiency of TAT-RasGAP_317-326_ towards *A. baumannii* was further increased by combining it with a selection of antibiotics. Combination with gentamicin or meropenem resulted in synergism towards planktonic bacteria and increased efficiency towards biofilm^17^.

In this study, we assessed the capacity of different *A. baumannii* strains to acquire resistance to TAT-RasGAP_317-326_ in vitro and determined whether the acquisition of resistance to this peptide caused the appearance of cross-resistance to other antimicrobial agents. We show that cross-resistance to polymyxins emerges without a strong impact on bacterial fitness and virulence. Polymyxins are non-ribosomal cationic peptides, having no sequence homology with TAT-RasGAP_317-326_. Cross-resistance is caused by the acquisition of mutations in the genes encoding the PmrAB two-component system. Some mutations we detected are identical to documented mutations found in clinical strains of *A. baumannii* resistant to polymyxins. These results indicate that treatment with an AMP, which amino acid sequence differs from polymyxins could still cause the emergence of resistance to polymyxins in *A. baumannii* and highlights the need for caution regarding the clinical application of AMPs.

## Results

### In vitro selection of resistance towards TAT-RasGAP_317-326_ in *A. baumannii* recurrently results in cross-resistance to polymyxins

**Fig. 1:**
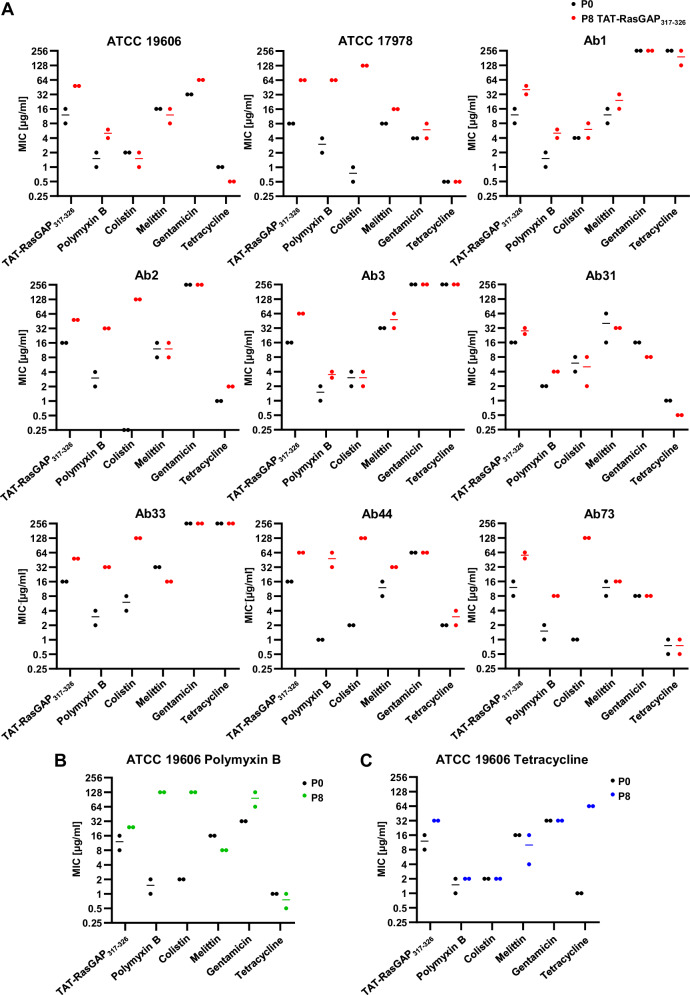
In vitro selection resistance in the presence of TAT-RasGAP_317-326_ can induce both specific resistance and cross-resistance to polymyxins in different strains of *Acinetobacter baumannii.* The indicated strains were incubated with increasing concentrations of TAT-RasGAP_317-326_ (**A**), polymyxin B (**B**), or tetracycline (**C**) for a total of eight passages to obtain a resistant isolate (P8). Minimal inhibitory concentrations of the indicated antimicrobial agents were determined for both P8 and parental strains (P0). Exact values are shown in Table 1 (P0), Table 2 (P8 with TAT-RasGAP_317-326_), and Table 3 (P8 selected with polymyxin B or tetracycline). Measurements were performed in biologically independent duplicates, and lines represent the average of the two values, when different.

In order to study the potential of *A. baumannii* isolates to develop resistance to TAT-RasGAP_317-326_, we selected nine isolates with diverse backgrounds and antibiotic resistance profiles, comprising two well-studied ATCC strains and seven clinical isolates (Table 1). The seven clinical isolates were selected from collections of isolates used in two former studies^16,19^ according to their resistance profiles, thus opting for isolates sensitive or resistant to classical antibiotics such as gentamicin and tetracycline and excluding extensive drug-resistant strains, in which further resistance selection would be hazardous. These strains were subjected to eight passages in liquid culture with increasing concentrations of the antimicrobial peptide TAT-RasGAP_317-326_, starting with subinhibitory concentrations and then increasing concentrations when growth was observed, similarly as was already performed with other bacterial species^20,21^. In parallel, the same experiment was performed on the ATCC 19606 strain using polymyxin B and tetracycline, respectively. Resistance profiles of the selected strains were then measured (Fig. 1 and Tables 2, 3). A limited but consistent increase in MIC of TAT-RasGAP_317-326_ was observed in a large majority of strains selected with this peptide (Fig. 1A). Interestingly, development of resistance to this peptide was linked, in some cases to the development of cross-resistance to polymyxins (polymyxin B and colistin), but not to the AMP melittin or to antibiotics gentamicin or tetracycline (Table 2, resistance development highlighted in bold). In contrast, incubation of the ATCC 19606 strain with increasing concentrations of polymyxin B or tetracycline induced the emergence of resistance specific to polymyxins or tetracycline, respectively (Fig. 1B, C and Table 3). We did not observe the appearance of any potent collateral sensitivity, as observed in other studies^7^.

**Table 1:** List of *A. baumannii* strains used in this study

Isolate name	MIC TAT	MIC PolB	MIC Col	MIC Mel	MIC Genta	MIC Tetra	Ref.
ATCC 19606	8–16	1–2	2	16	32	1	ATCC
ATCC 17978	8	2–4	0.5-1	8	4	0.5	ATCC
Ab1	8–16	1–2	4	8–16	>256	>256	Heulot
Ab2	16	2–4	0.25	8–16	>256	1	Heulot
Ab3	16	1–2	2–4	32	>256	>256	Heulot
Ab31	16	2	4–8	16–64	16	1	Leshkasheli
Ab33	16	2–4	4–8	32	>256	>256	Leshkasheli
Ab44	16	1	2	8–16	64	2	Leshkasheli
Ab73	8–16	1–2	1	8–16	8	0.5– 1	Leshkasheli

MICs of TAT-RasGAP_317-326_ (TAT), polymyxin B (PolB), colistin (Col), melittin (Mel), gentamicin (Genta), and tetracycline (Tetra) against the indicated strains were measured in biologically independent duplicates. Both values are indicated when different results were obtained.

**Table 2:** MICs of strains selected with TAT-RasGAP_317-326_

Isolate name	MIC TAT	MIC PolB	MIC Col	MIC Mel	MIC Genta	MIC Tetra
ATCC 19606	**48**	4–6	1–2	8–16	64	0.5
ATCC 17978	**64**	**64**	**>128**	16	4–8	0.5
Ab1	**32–48**	4–6	4–8	16–32	>256	128–256
Ab2	**48**	**32**	**128**	8–16	>256	2
Ab3	**64**	3–4	2–4	32–64	>256	256
Ab31	24–32	4	2–8	32	8	0.5
Ab33	**48**	**32**	**>128**	16	>256	>256
Ab44	**64**	**32–64**	**>128**	32	64	2–4
Ab73	**48–64**	**8**	**128**	16	8	0.5–1

MICs of TAT-RasGAP_317-326_, polymyxin B, colistin, melittin, gentamicin, and tetracycline against the indicated strains were measured in biologically independent duplicates. Both values are indicated when different results were obtained. Bold values indicate that the measured MICs are at least three times higher than the parental strain.

**Table 3:** MICs on the strains selected with polymyxin B or tetracycline

Isolate name	MIC TAT	MIC PolB	MIC Col	MIC Mel	MIC Genta	MIC Tetra
ATCC 19606 PolB	24	**128**	**>128**	8	64–128	0.5–1
ATCC 19606 Tetra	32	2	2	4–16	32	**64**

MICs of TAT-RasGAP_317-326_, polymyxin B, colistin, melittin, gentamicin, and tetracycline against the indicated strains were measured in biologically independent duplicates. Both values are indicated when different results were obtained. Bold values indicate that the measured MICs are at least three times higher than the parental strain.

### Development of resistance towards TAT-RasGAP_317-326_ does not cause collateral fitness defects

**Fig. 2:**
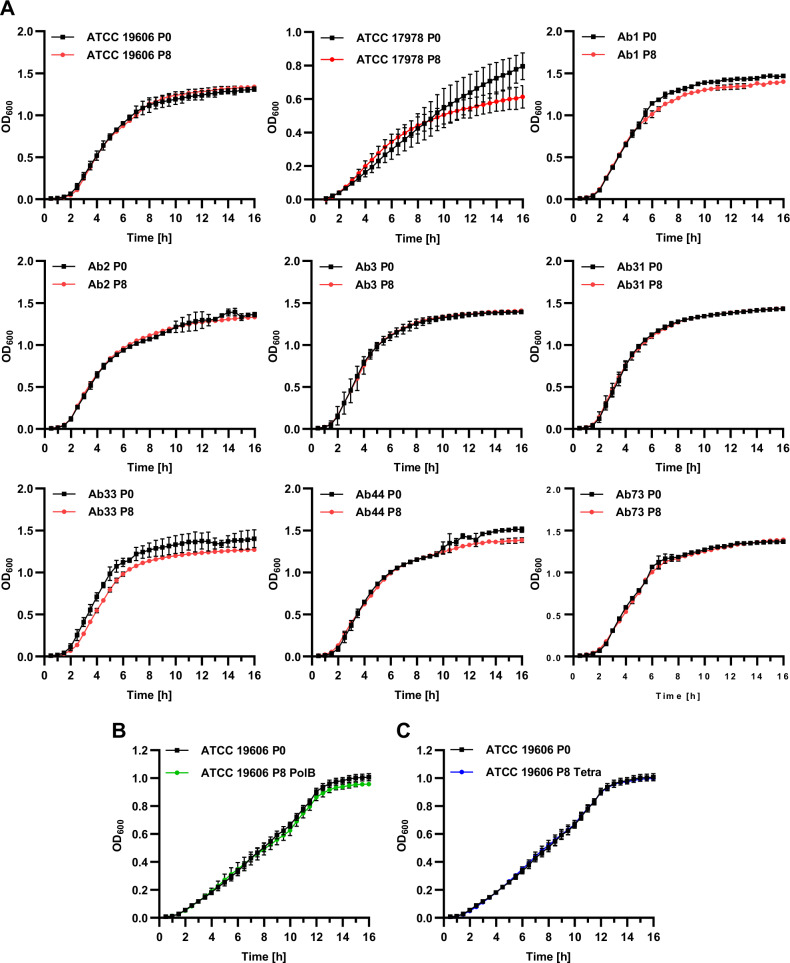
Resistance acquisition does not induce in vitro growth defects. P0 and P8 *A. baumannii* isolates upon selection with TAT-RasGAP_317-326_ (**A**), polymyxin B (**B**), or tetracycline (**C**) were grown overnight in LB and diluted to an OD_600_ of 0.01 in fresh medium. Growth was then assessed by OD_600_ measurement each 30 min for a total of 16 h. Values are the average of three experiments. Error bars represent the standard deviation of the three replicates.

Acquisition of resistance is sometimes linked to a fitness cost. We thus measured the growth rate of resistant mutants and compared it with the growth of the parental strains. We did not observe any growth defect, both in LB (Fig. 2) and in LB supplemented with NaCl or glucose (Figs. S1, S2). Moreover, we did not observe any important morphological changes of planktonic bacteria between the resistant isolates and their parental counterparts (Fig. S3).

**Fig. 3:**
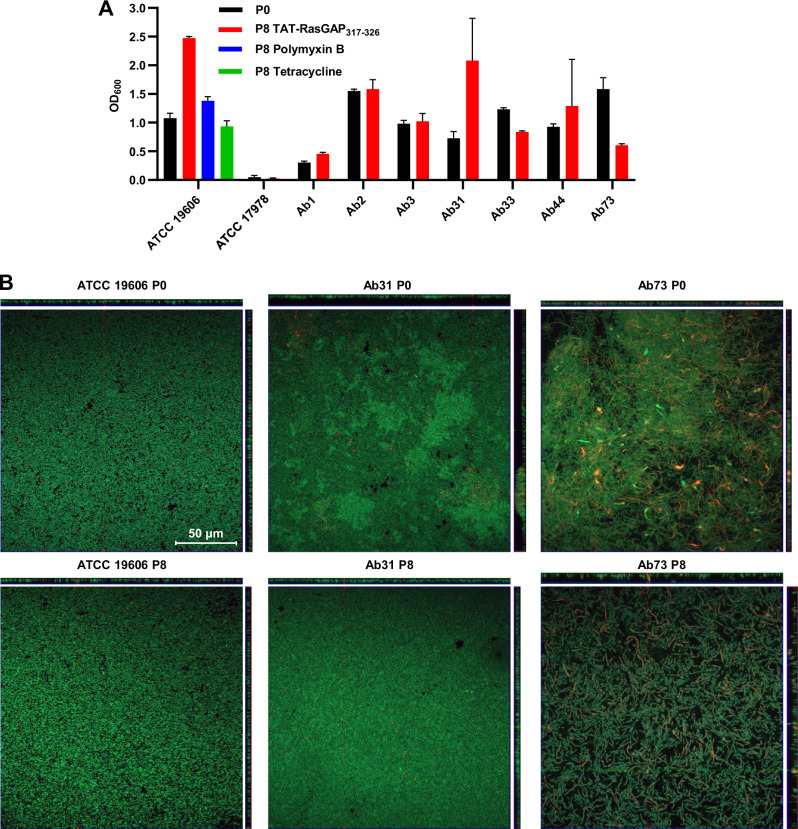
Biofilm formation and virulence are not strongly affected in *A. baumannii* isolates resistant to TAT-RasGAP_317-326_. **A** In vitro biofilm formation was assessed for both P0 and P8 isolates, by quantifying biofilm biomass through crystal violet staining. The average of biologically independent duplicates are presented. Error bars show standard deviation. **B** The effect of resistance acquisition on the structure of the biofilm was assessed by live-dead staining followed by Z-stacks acquisition with a confocal microscope. Reconstruction of Z projections are shown on the top and on the right for each strain. Three representative strains are shown.

Since *A. baumannii* is well known to form biofilms, we decided to test whether the development of resistance affects the capability of the different strains to form biofilms, using an in vitro model we already used in the past^17^. Using crystal violet staining, we quantified the amount of biofilm formed by the different resistant strains and compared them with their parental counterparts (Fig. 3A). We did not observe clear trends when comparing resistant and sensitive strains. In a further step, we formed biofilms on glass coverslips, labeled them with live-dead staining and performed confocal microscopy (Z-stacks) in order to observe the morphology and thickness of the different biofilms (Fig. 3B and Fig. S4). Again, these observations indicated that acquisition of resistance did not induce defects in biofilm formation in the *A. baumannii* strains, in the conditions we tested.

Taken together, these results indicate that the acquisition of both resistance specific to TAT-RasGAP_317-326_ and cross-resistance to polymyxins do not visibly influence bacterial fitness and morphology.

### Emergence of cross-resistance does not depend on a specific genetic background

We observed that cross-resistance to polymyxins emerged only in five of the nine strains we tested. Since the resistance selection was performed only once per strain, it was impossible at that stage to determine whether cross-resistance can emerge only in a subset of strains. We thus repeated resistance selection for all the strains in triplicate. We measured the MICs of TAT-RasGAP_317-326_ and polymyxin B on the resulting strains (Table 4 and Fig. S5). We could detect an increase in the MIC of TAT-RasGAP_317-326_ (more than threefold increase) upon selection in 13 of the 27 selected isolates (Table 4, highlighted in bold). In contrast, the increase of MIC of TAT-RasGAP_317-326_ upon resistance selection was less than threefold in the other replicates, indicating that the efficiency of resistance emergence can vary. Selection of resistance to TAT-RasGAP_317-326_ was again linked, in 16 of the 27 isolates, to cross-resistance towards polymyxin B. This appears to occur stochastically, independently of the strain genetic background, since cross-resistance was detected at least once in all strains apart from Ab31. It should be noted that in both experiments, we were not able to obtain a robust MIC increase in the Ab31 strain, indicating that this strain may have lower capabilities to become resistant to TAT-RasGAP_317-326_. Furthermore, in order to determine whether resistance acquisition was stable, we passaged ATCC 19606 resistant isolates for a total of eight times in the absence of any antimicrobial agent. Resistance to polymyxins was unchanged after these 8 passages, indicating that resistance could not be easily lost (Fig. S6).

**Table 4:** MICs of strains selected with TAT-RasGAP_317-326_

Isolate name	MIC TAT-RasGAP_317-326_			MIC Polymyxin B		
Replicate	b	c	d	b	c	d
ATCC 19606	16–64	**32–64**	**32–64**	4–6	**64**	2–4
ATCC 17978	16–32	**64–96**	**48–64**	0.5–1	**32–64**	0.5
Ab1	**128-** > **128**	**64**	32	**128-** > **128**	**>128**	4
Ab2	**32–64**	16–64	**64**	1–2	**64**	**64**
Ab3	16–32	32	**64**	4–8	**32**	**128-** > **128**
Ab31	24	24–32	16–32	2–4	**64–128**	4–8
Ab33	**48**	**128**	**48**	**>128**	**128-** > **128**	**>128**
Ab44	24–32	32	**64**	**16**	**32**	**128-** > **128**
Ab73	12	12–16	16	4	2	**32–64**

MICs of TAT-RasGAP_317-326_ and polymyxin B against the indicated strains were measured in biologically independent duplicates. Both values are indicated when different results were obtained. Values in bold indicate that the measured MICs are at least three times higher than the parental strain.

### Cross-resistance emergence is generally linked to mutations in *pmrAB*

In order to better understand the mechanisms involved in the acquisition of cross-resistance, we performed whole genome sequencing on the resistant strains obtained in the first experiment (Table 2) and compared their genome with the corresponding parental strains. The identified mutations resulting in amino acid changes are shown in Table S1. Some of the detected mutations in isolates specifically resistant to TAT-RasGAP_317-326_ affect outer membrane proteins (OmpA, BauA), while others might influence gene expression regulation (TopA). Strain Ab1 is potentially a hypermutator strain since it acquired up to 20 mutations, while others only acquired between 1 and 4 mutations (Table S1).

Interestingly, four isolates out of the five that developed cross-resistance to polymyxin B acquired a mutation in the *pmrAB* operon. This operon encodes the two-component system PmrAB, which regulates LOS modifications, in particular the addition of the cationic sugar L-4-aminoarabinose to the lipid A phosphate group of LOS^14^. Mutations in PmrAB were already identified in polymyxin-resistant clinical isolates^22^.

**Fig. 4:**
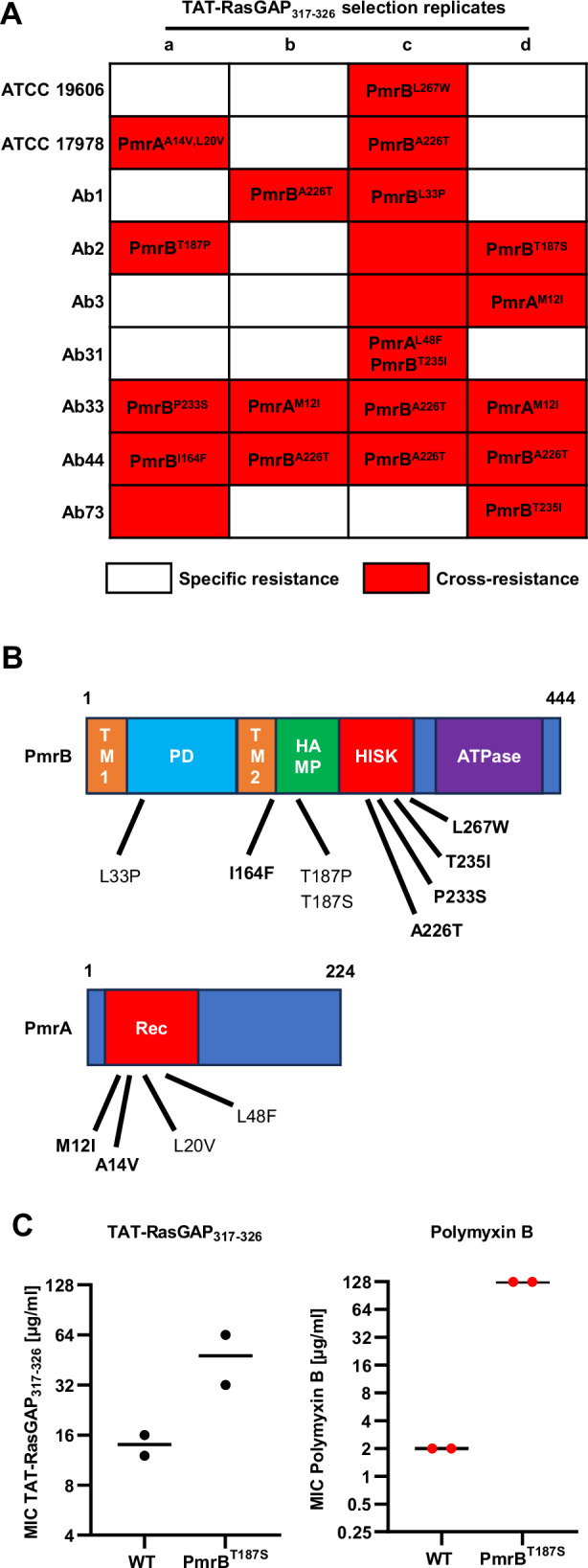
Emergence of cross-resistance to polymyxins upon selection with TAT-RasGAP_317-326_ mostly occurs through point mutations in *pmrAB.* **A** Resistant isolates depicted in Tables 2, 4 are classified as bearing a specific resistance (white) or cross-resistance (red). Sequencing of *prmA* and *pmrB* genes was performed on all strains, and mutations found upon comparison with the parental strains are shown. **B** Amino acid changes caused by mutations found in cross-resistant strains were mapped on the protein domains of PmrA and PmrB. Mutations already described in the literature are highlighted in bold. **C** The PmrB^T187S^ mutation is sufficient to cause cross-resistance to TAT-RasGAP_317-326_ and polymyxin B. Targeted mutagenesis using a CRISPR-Cas9-based method was performed in an ATCC 17978 wild-type strain. MICs of the indicated antimicrobial agents towards the mutant and the wild-type strain were measured in biologically independent duplicates. Black lines represent the average of the replicates.

To further confirm the implication of the PmrAB two-component system in cross-resistance, we amplified *pmrA* and *pmrB* by PCR from all the resistant strains obtained and performed Sanger sequencing to identify potential mutations in these two genes. We detected mutations in *pmrA* or *pmrB* in 18 of the 21 cross-resistant isolates. In comparison, none of the specific resistant isolates had any mutations in these two genes (Fig. 4A). We then mapped the mutations we detected on PmrA and PmrB protein domains and searched the literature to verify whether some of these mutations were already described. Indeed, 7 out of the 11 mutated sites we detected have already been found in clinical *A. baumannii* isolates resistant to colistin^22^ (Fig. 4B, mutations already described in literature are highlighted in bold).

### Mutation in *pmrB* is sufficient to cause cross-resistance to TAT-RasGAP_317-326_ and polymyxin B

In order to confirm that such mutations are indeed the cause of the cross-resistance to TAT-RasGAP_317-326_ and polymyxin B, we used a CRISPR-Cas9-based method to insert a single mutation in a wild-type background^23^. Using this method, we introduced the T187S mutation in the ATCC 17978 strain, which was sufficient to cause an increase in MIC of both TAT-RasGAP_317–326_ and polymyxin B towards this strain (Fig. 4C).

### Cross-resistance sometimes appears without mutations in *pmrAB*

In three cross-resistant isolates, the phenotype could not be attributed to mutations in *pmrAB* (Ab2c, Ab3c, Ab73a). We already had the genome sequence of Ab73a (Table S1). We thus performed whole genome sequencing of the two isolates, Ab2c and Ab3c, and identified mutations by comparing them with their respective parental strains (Table S2).

Four mutations could be detected in strain Ab73a. Two of them caused a change of one amino acid in the proteins BamA and LptC, respectively. A third caused the appearance of a premature stop codon, producing a truncated form of PlcD, and the last one caused a frameshift in the gene encoding PldA. We recently identified a potential role for the essential outer membrane insertase BamA as a receptor for TAT-RasGAP_317-326_ in *E. coli*^24^. LptC is a periplasmic protein of the LOS transporter machinery. PlcD and PldA are phospholipases. Interestingly, inhibition of phospholipase function was shown to improve fitness of a LOS-deficient mutant in *A. baumannii*^25^. It is thus possible that inactivation of phospholipases in the Ab73a strain compensates for some defect in LOS transport caused by the LptC mutation.

A total of ten mutations was detected in the Ab2c strain, mainly affecting uncharacterized proteins. However, some detected point mutations caused amino acid changes in UvrA, a thiamine permease, and BasI, an acinetobactin biosynthetic protein. Finally, the start codon of the *crp* gene, encoding the cyclic-AMP receptor protein, was mutated. This is expected to induce a depletion of the Crp protein. Crp is an important transcriptional regulator (regulating over 180 genes in *E. coli*^26^), which is regulated by cyclic-AMP binding. Cyclic-AMP has recently been identified as an important global virulence regulator in *A. baumannii*^27^. Depletion of Crp might thus strongly affect cellular properties and thus lead to a cross-resistance phenotype.

Concerning the Ab3c strain, only three single point mutations were detected in two genes, encoding uncharacterized proteins: a AAA family ATPase and an integrase family protein. Because these two proteins are uncharacterized, we cannot explain their role in cross-resistance. Overall, PmrAB-independent cross-resistance phenomena need to be further investigated in the future.

### Emergence of cross-resistance does not affect bacterial virulence

**Fig. 5:**
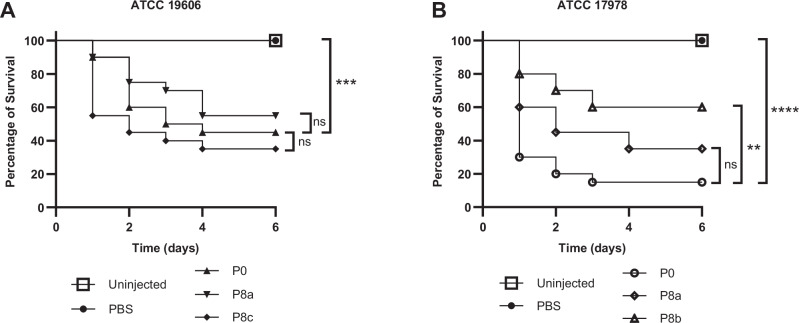
Development of resistance does not strongly affect *A. baumannii* virulence. Virulence of resistant isolates was compared to parental strains in a *Galleria mellonella* model. 10^6^ bacteria of the indicated strains were resuspended in 20 µl of PBS and were injected into the posterior proleg of 20 larvae for each strain. Injection of PBS alone was used as a control. Survival was assessed at the indicated time points. This was performed in ATCC 19606 (**A**) and ATCC 17978 (**B**) backgrounds in triplicate. Representative results are shown. GraphPad Prism was used to test for significant differences between the curves using the log-rank (Mantel-Cox) test. Significance levels are indicated by asterisks (**p value <0.01, ***p value <0.001, ****p value <0.0001).

Since we observed no consistent influence of cross-resistance development on bacterial fitness in vitro, we decided to take advantage of the specific and cross-resistant isolates we obtained in the same background to assess their virulence in an in vivo *Galleria mellonella* model. We decided to limit our study to the well-described ATCC 19606 and ATCC 17978 strains. Indeed, infection models of *G. mellonella* using these two strains have been described in the literature^28,29^. We thus infected groups of *G. mellonella* larvae with equivalent quantities of *A. baumannii* (10^6^ bacteria per larva), either sensitive (P0), specifically resistant to TAT-RasGAP_317-326_ (ATCC 19606 P8a and ATCC 17978 P8b), or cross-resistant to TAT-RasGAP_317–326_ and polymyxins (ATCC 19606 P8c and ATCC 17978 P8a). Injection of all strains caused a significant drop in *G. mellonella* viability in comparison to the PBS injected control (Fig. 5). No significant difference was observed between the different ATCC 19606 isolates (Fig. 5A). A limited but significant increase in viability was observed when infection was performed with the ATCC 17978 P8b strain, which shows specific resistance to TAT-RasGAP_317-326_ (Fig. 5B). Taken together, these results do not show a significant effect of the acquisition of cross-resistance on *A. baumannii* virulence. However, virulence might be lowered in some cases by acquisition of specific resistance to TAT-RasGAP_317-326_.

## Discussion

AMPs have been identified as promising antimicrobial agents towards multidrug-resistant (MDR) bacteria. Despite the recent progress made towards their clinical application, little is known about how pathogenic bacteria would adapt to a broad clinical usage of AMPs. In this study, we investigated how one of the most successful nosocomial pathogens, *Acinetobacter baumannii*, adapts in vitro to the AMP TAT-RasGAP_317-326_. *A. baumannii* is known to rapidly evolve resistance and indeed clinical isolates are commonly MDR. In this study, we showed that *A. baumannii* can acquire resistance to TAT-RasGAP_317-326_. Importantly, in approximately half of the cases, we could observe the emergence of cross-resistance to polymyxins. Since polymyxins are used as last-resort antibiotics towards infections caused by MDR bacteria, the appearance of such cross-resistance is highly problematic. In addition, we did not observe any significant reduction in bacterial fitness and virulence linked to the emergence of cross-resistance. This indicates that the fitness cost induced by cross-resistance emergence is negligible. Nevertheless, the tests we performed were done in vitro (growth tests and biofilm formation) and in a simple *G. mellonella* in vivo model. We thus cannot rule out that cross-resistance may still influence biofilm formation or virulence in more challenging conditions, as found during human infection.

In a large majority of cross-resistant isolates, mutations in *pmrAB* genes were detected. Even though resistance selection towards TAT-RasGAP_317-326_ was performed in vitro, some of the mutations we obtained were identical to mutations detected in polymyxin-resistant clinical isolates (Fig. 4B, in bold). Resistance to polymyxins can be caused by the addition of phosphoethanolamine to lipid A. This reduces the negative charge of LPS and its affinity to polymyxins^13^. Mutations in genes encoding the PmrAB two-component system result in the overexpression of *pmrC*, which encodes a phosphoethanolamine transferase^14^. Most of the mutations detected in clinical isolates were located in the histidine kinase domain of PmrB (HISK), which is involved in autophosphorylation and activation of PmrA^30^. Similarly, we detected in our study several mutations targeting the HISK domain (Fig. 4B). Other mutations obtained are localized in the periplasmic domain (PD), the second transmembrane domain (TM2) and in the Histidine kinases, Adenylyl cyclases, Methyl-binding proteins, and Phosphatases domain (HAMP, Fig. 4B). How these mutations increase the activity of PmrAB is not well understood, however mutations in these domains were also found in clinical isolates (I164F for example)^31^. Finally, mutations detected in PmrA were all localized in the receiver domain (Rec), which was shown to be the main domain where mutations were detected in clinical isolates^22^. Selection of such mutations indicates that LPS may mediate the affinity of both TAT-RasGAP_317-326_ and polymyxins with *A. baumannii*. This is consistent with the fact that both TAT-RasGAP_317-326_ and polymyxins are cationic peptides, which may interact with the negatively charged LPS. However, this cross-resistance does not extend to all cationic AMPs: mutants cross-resistant to TAT-RasGAP_317-326_ and polymyxins are not resistant to melittin. More experiments need to be done now with a broader set of AMPs and should allow to differentiate their modes of interaction with the bacterial surface.

Another interesting observation is the emergence of cross-resistance in ~50% of the isolates. This indicates that several mechanisms of resistance towards TAT-RasGAP_317-326_ can be selected, and only a subset of those results in cross-resistance to polymyxins. We observed the emergence of cross-resistance at least once in each strain background we tested, indicating that this process is strain-independent. However, the rate of cross-resistance emergence seems to vary in different strains (from one to four cross-resistant isolates, depending on the strain, Fig. 4A). This might indicate that the rate of emergence of cross-resistance varies according to the strain background. We thus aim in the future at repeating resistance selection and increasing the number of replicates to be able to assess whether some strains are more prone than others to develop cross-resistance and possibly link this with other specific properties of the strains, such as spontaneous mutation rate, which was shown to vary in clinical isolates of *A. baumannii*^32^. Nevertheless, we observed no clear difference between cross-resistance and specific resistance regarding their influence on fitness and virulence. This would explain why both can occur at a similar rate.

In a limited number of cases, cross-resistance was observed in the absence of mutations in *pmrAB* genes. In these cases, whole genome sequencing allowed us to identify mutations possibly involved in cross-resistance development. Most promising candidates are a point mutation in *lptC*, encoding a LPS transporter subunit, and a mutation of the start codon of *crp*, encoding a cyclic-AMP receptor protein. LptC is an essential LPS transporter subunit and is the target of antimicrobial agents such as thanatin^33^. It was identified as a member of the polymyxin B resistome in *Pseudomonas aeruginosa*^34^. The acquired mutation (V146E, Table S1) occurs in the periplasmic domain of LptC (β12), changing a highly conserved valine to a glutamic acid^35^. No similar mutation could be found in the literature. The mutation is not localized at the interface between LptA and LptC, which is targeted by thanatin^33^. This mutation may, however, modify the conformation of LptC and thus induce changes in the efficiency of LPS transport to the outer membrane, causing changes in the affinity of polymyxin B and TAT-RasGAP_317-326_ to bacterial cells. CRP is a transcriptional regulator that modulates the expression of a large number of genes^26^. It was shown to be involved in antimicrobial resistance in diverse bacterial species by modulating the stress response, by impacting LPS modifications or through modulation of the membrane potential^36–38^. Recently, a link between CRP, LPS modifications, and polymyxin resistance was highlighted in *E. coli*^39^. CRP may thus play a similar role in *A. baumannii*. However, the exact roles of mutations in *lptC* and *crp* in cross-resistance in *A. baumannii* need to be investigated in the future.

Despite several attempts, we never obtained cross-resistance in another Gram-negative bacterium, *Escherichia coli*. Previous attempts were performed using the MG1655 strain, whose LPS does not contain O-antigen. This incomplete LPS might hinder the development of cross-resistance. Alternatively, it could be that the genome plasticity of *E. coli* is lower, making mutations less frequent. This may cause lower diversity of the outcomes during resistance selection. This needs to be investigated, through artificially increasing the mutational rate of *E. coli* via mutagenic agents, for example. *E. coli* strains synthesizing a complete LPS containing the O-antigen could also be studied in order to determine what factors make cross-resistance development less favorable in *E. coli*.

Taken together, our results show the strong adaptability of bacteria towards antimicrobial agents. This adaptability can lead to the development of cross-resistance that can be highly deleterious for the clinical usage of some antimicrobial agents. Furthermore, it was recently shown that ESKAPE pathogens can rapidly develop resistance towards newly developed antibiotics in vitro^40^. This needs to be considered before any new antimicrobial agent is released and used broadly in clinical settings. Broad range systematic mapping of cross-resistance events could help us to determine which agents are less prone to develop cross-resistance^41^. Modifications of the AMP sequence, as well as further modifications (use of peptidomimetics, cyclization, and lipidation)^42^ may lead to the development of AMPs with lower resistance rates. In addition, combinations between drugs, for which no cross-resistance was observed, may be promising for innovative treatment strategies. This may lead bacteria into an evolutionary dead-end, the bacteria being unable to develop resistance to both antimicrobial agents. Development of modified AMPs and selection of the ideal combinations may be in the future assisted by artificial intelligence, allowing to predict the most promising treatment strategies^43^. Finally, appropriate dosage and use of the most efficient antimicrobial agents may also help avoid the emergence of unwanted resistance.

## Material and methods

### Bacterial strains, culture conditions, and antimicrobial agents

All strains used in this study are listed in Table S3. Bacteria were grown overnight at 37 °C with 200 rpm shaking in LB medium (10 g/L tryptone, 5 g/L yeast extract, and 10 g/L NaCl), or statically on an LB agar plate (LB supplemented with 15 g/L agar). TAT-RasGAP_317-326_ retro-inversed antimicrobial peptide (amino acid sequence DTRLNTVWMWGGRRRQRRKKRG) was provided by SBS Genetech (Beijing, China). Polymyxin B, apramycin, and spectinomycin were from Sigma-Aldrich brand (Merck, Darmstadt, Germany), gentamicin, tetracycline, and kanamycin by Applichem GmbH (Darmstadt, Germany), melittin by Enzo Life Science (Farmingdale, NY, USA), and colistin by LGC (Teddington, UK).

### Minimal inhibitory concentration (MIC) measurements

Overnight cultures were diluted to OD_600_ = 0.1 in fresh LB and incubated for 1 h at 37 °C with shaking. Cultures were then diluted 10x, and 10 µl of it was exposed to twofold serial dilutions of antimicrobials in LB in 96-well plates (in a total of 100 µl per well). After an incubation of ~18 h at 37 °C in static conditions and a humidified atmosphere, OD_600_ was measured using a FLUOstar Omega microplate reader (BMG Labtech, Ortenberg, Germany). The MIC was defined as the lowest concentration of the antimicrobial causing at least a 90% decrease in bacterial density. Measurements were performed in independent biological duplicates and were always performed in parallel with the parental strain.

### In vitro resistance selection

Overnight cultures were diluted 100x in 500 µl of LB containing 0.5 MIC of the indicated antimicrobial agent and incubated under shaking at 37 °C overnight. Similar dilution was then performed in medium containing the same concentration of antimicrobial agent or a higher concentration. Culture in which bacterial growth could be detected was further diluted similarly as before. This was repeated for a total of eight passages.

### Growth curves

Overnight cultures were diluted in fresh LB, LB with 0.4% glucose (10 g/L tryptone, 5 g/L yeast extract, 4 g/L glucose, 10 g/L NaCl), or LB with 2% NaCl (10 g/L tryptone, 5 g/L yeast extract, and 20 g/L NaCl) to obtain an OD_600_ = 0.01. 250 µl of the bacterial suspension was transferred to a 96-well polypropylene plate. The OD_600_ was measured every 30 min during 16 h at 37 °C with shaking using a BioTek, EPOCH 2 microplate spectrophotometer (Agilent, Santa Clara, CA, USA).

### Microscopy

Overnight cultures were diluted to an OD_600_ of 0.1 and incubated for 2 h at 37 °C. Cultures were then centrifuged at 5000 rpm for 2 min, supernatant was discarded, and bacterial pellet was resuspended in the remaining medium. 5 µl of bacterial suspension was then mounted on a microscopy slide. Pictures were taken using a light AXIO Imager 2 microscope (Zeiss, Oberkochen, Germany) with a 100x oil immersion objective and the ZEN software. Images were then treated using ImageJ software^44^.

### Whole genome sequencing and Sanger sequencing

Genomic DNA was extracted from overnight cultures using the Wizard genomic purification kit (Promega, Madison, WI) and quantified using the Qubit system (Thermo Fisher Scientific, Waltham, MA, USA). A Nextera XT Kit (Illumina, San Diego, CA) was then used to produce libraries, whose quality was controlled by Fragment Analyzer AATI (Agilent). Sequencing was performed on a MiSeq system using MiSeq Reagent Kits v2 (Illumina). Reads were assembled with spades v. 3.11.1^45^ and mapped on the corresponding reference genomes with bwa v. 0.7.17^46^. Variant calling was performed using gatk 4.0.2.0^47^. Identified SNPs were manually checked by visualizing the mapping with JBrowse^48^.

Verification of mutations in specific genes was performed by amplification of the gene of interest by colony PCR. Primers upstream and downstream of the region of interest (Listed in Table S3) were designed using Geneious (Biomatters, Auckland, New Zealand), and PCR mix was prepared by combining 12.5 µl of 2x Taq MasterMix Dye (Promega) and 1 µl of 10 µM dilutions of each primer, in a total volume of 25 µl. A single colony of the strain of interest was resuspended in the mix and PCR protocol in a Biometra TRIO Thermocycler (Labgene Scientific, Châtel-Saint-Denis, Switzerland) using the following parameters: 95 °C 5 min; 40 cycles of 95 °C 30 s, 54 °C 30 s, 72 °C (1 min for every kbp of product); and finally, 72 °C for 10 min. Proper amplification and correct size of PCR products were checked on a 1% agarose gel (120 V, 400 amp, 20 min). PCR products were purified using a QIAquick PCR purification system (Qiagen, Hilden, Germany) and sent to Microsynth (Balgach, Switzerland) for Sanger sequencing. Resulting sequences were aligned to the reference gene using Geneious software.

### Biofilm formation assays

Biofilm formation assays were mostly performed as described earlier^18^. Overnight bacterial cultures were diluted 1:50 into fresh LB and further incubated for 4 h. Bacteria were then washed two times with PBS pH 7.4 and resuspended in freshly prepared BM2 medium (62 mM potassium phosphate buffer, 7 mM ammonium sulfate, 10 µM iron sulfate, 40% glucose, 50% casein peptone, 2 mM magnesium sulfate) to obtain a final OD_600_ of 0.1. 100 µl of bacterial suspension was then transferred into a 96-well polypropylene plate and 250 in 24-well plates containing glass coverslips. The plate was incubated 24 h at room temperature in humidified atmosphere. Twenty-four-well plates were tilted at a 45° angle during the whole incubation time. For biomass quantification, the 96-well polypropylene plate was washed two times with nanopure H_2_0, and 125 µl of 0.1% crystal violet (Sigma, V5265) was added into each well. After 10 min incubation, the plate was washed again three times with water, and dried overnight. Accumulated dye was then resuspended with 30% acetic acid for 10 min and transferred into a new 96-well plate. Absorbance at 600 nm was then measured using a FLUOstar Omega microplate reader. The 24-well polypropylene plates were washed two times with PBS and labeled with LIVE/DEAD BacLight Kit (Invitrogen) following the manufacturer’s instructions for 15 min in the dark. Following two washes with PBS, bacteria were fixed with 4% paraformaldehyde, washed two more times with PBS, and mounted on a glass slide using Moewiol resin. Biofilms were imaged using the Z-stacks option of a confocal microscope (LSM900, Zeiss) with a 40x oil immersion objective. Orthogonal projections were reconstructed using the ZEN software version 3.0.

### CRISPR-Cas9-based targeted mutagenesis

Point mutations were inserted in the genome of *A. baumannii* strain ATCC 17978 using the CRISPR-Cas9 genome editing method described by Wang et al.^23^ with some adaptations and optimizations. All oligonucleotides used in this experiment were designed using Geneious software, synthesized by Microsynth AG, and are listed in Table S3. Forward and reverse oligonucleotides encoding the desired gRNA (1 µl of Spacer-F and Spacer-R) were phosphorylated with a T4 polynucleotide kinase (New England BioLabs, Ipswich, MA, USA) in a total volume of 50 µl for 1 h at 37 °C. About 0.5 µl of NaCl 5 M was added to the phosphorylated product, and annealing was performed by incubating the sample at 95 °C for 5 min, followed by a gradual decrease in temperature at a rate of 1 °C per 10 s until reaching 25 °C. Cloning of the gRNA encoding sequence in the pSGAb-km expression plasmid was performed using Goldengate assembly with 1 µl of 20-fold diluted annealed oligonucleotides, 50 ng of plasmid, 0.5 µl of T4 DNA ligase and 0.5 µl of Bsal HFv2 (New England Biolabs) in a total volume of 10 µl. Goldengate assembly was performed using a thermocycler with the following program: 25 cycles (3 min at 37 °C and 4 min at 16 °C) followed by 5 min at 50 °C and 10 min at 80 °C. About 5 µl of the product was then transformed into thermocompetent DH5α, and colonies were screened by PCR using the M13R and Spacer-F primers. The plasmid containing the correct insert was then harvested by miniprep (GeneJET Plasmid Miniprep Kit, Thermo Fisher Scientific). An overnight culture of *A. baumannii* ATCC 17978 strain baring the pCasAb-apr plasmid was diluted 100x into 50 mL of LB with apramycin and incubated at 37 °C until OD_600_ reached 0.1–0.15. Cas9 expression was induced by the addition of 1 mM IPTG (Applichem), and the culture was further incubated for 2 h. Bacteria were then washed three times with ice-cold MilliQ water and electroporation was performed with 100 or 200 ng of the pSGAb-km plasmid and 3 or 6 µl of the donor ssDNA (100 µM) using chilled 1 mm electroporation cuvette and a Gene Pulser Xcell Electroporation system (BioRad, Hercules, CA, USA). After electroporation, cells were immediately resuspended into 1 mL of ice-cold LB and incubated 1 h at 37 °C with shaking. Transformed cells were plated on LB agar plates supplemented with the corresponding antibiotics (apramycin and kanamycin) and incubated overnight. The gene of interest was amplified by colony PCR. The PCR product was purified with a QIAquick PCR purification kit (Qiagen) and sent for Sanger sequencing (Microsynth).

### Galleria mellonella infection model

Overnight cultures of the indicated *A. baumannii* strains were washed with PBS and serially diluted and plated on LB agar petri dishes. The number of living bacteria per ml was measured by Colony Forming Units quantification. OD_600_ of the culture was measured in parallel, and CFU/OD_600_ was then calculated. Bacteria were diluted to obtain a final suspension of bacteria corresponding to 10^6^ bacteria in 20 μl of PBS. 20 μl of the suspension were then injected into the posterior proleg of *Galleria mellonella* (Bait Express, Basel, Switzerland). Twenty larvae were injected per group, with control groups being either uninjected or injected with PBS. Larvae were then incubated at 37 °C, and survival was monitored daily up to 6 days.

### Statistical analyses

Averages and standard deviations between replicates, as well as statistical analyses on survival graphs (Mantel-Cox tests) were performed using GraphPad Prism software (La Jolla, CA).

## Supplementary information

Supplementary information

## Data Availability

All raw data acquired and used to produce the Figures and Tables presented in this article are either provided as supplementary data or can be made available upon request sent to the corresponding author. Sequences of the isolates used in this study were deposited in the European Nucleotide Archive with the project number PRJEB88265 (https://www.ebi.ac.uk/ena/browser/view/PRJEB88265).
