# Supplementary material for: Cross resistance emergence to polymyxins in Acinetobacter baumannii exposed in vitro to an antimicrobial peptide
Source: NPJ Antimicrob Resist. 2025 May 29;3:44. doi: 10.1038/s44259-025-00120-4 (PMC12122810; doi:10.1038/s44259-025-00120-4)
Supplement: Supplementary file 1 — Supplementary information [file 44259_2025_120_MOESM1_ESM.pdf]

**Figure S1. Resistance acquisition does not induce in vitro growth defects in presence of glucose.** P0 and P8 isolates upon selection with TAT-RasGAP$_{317-326}$ **(A)**, polymyxin B **(B)** or tetracycline **(C)** were grown overnight in LB and diluted to an $OD_{600}$ of 0.01 in fresh LB supplemented with 0.4% glucose. Growth was then assessed by $OD_{600}$ measurement each 30 minutes for a total of 16 hours. Values are the average of three experiments. Error bars represent standard deviation of the three replicates.

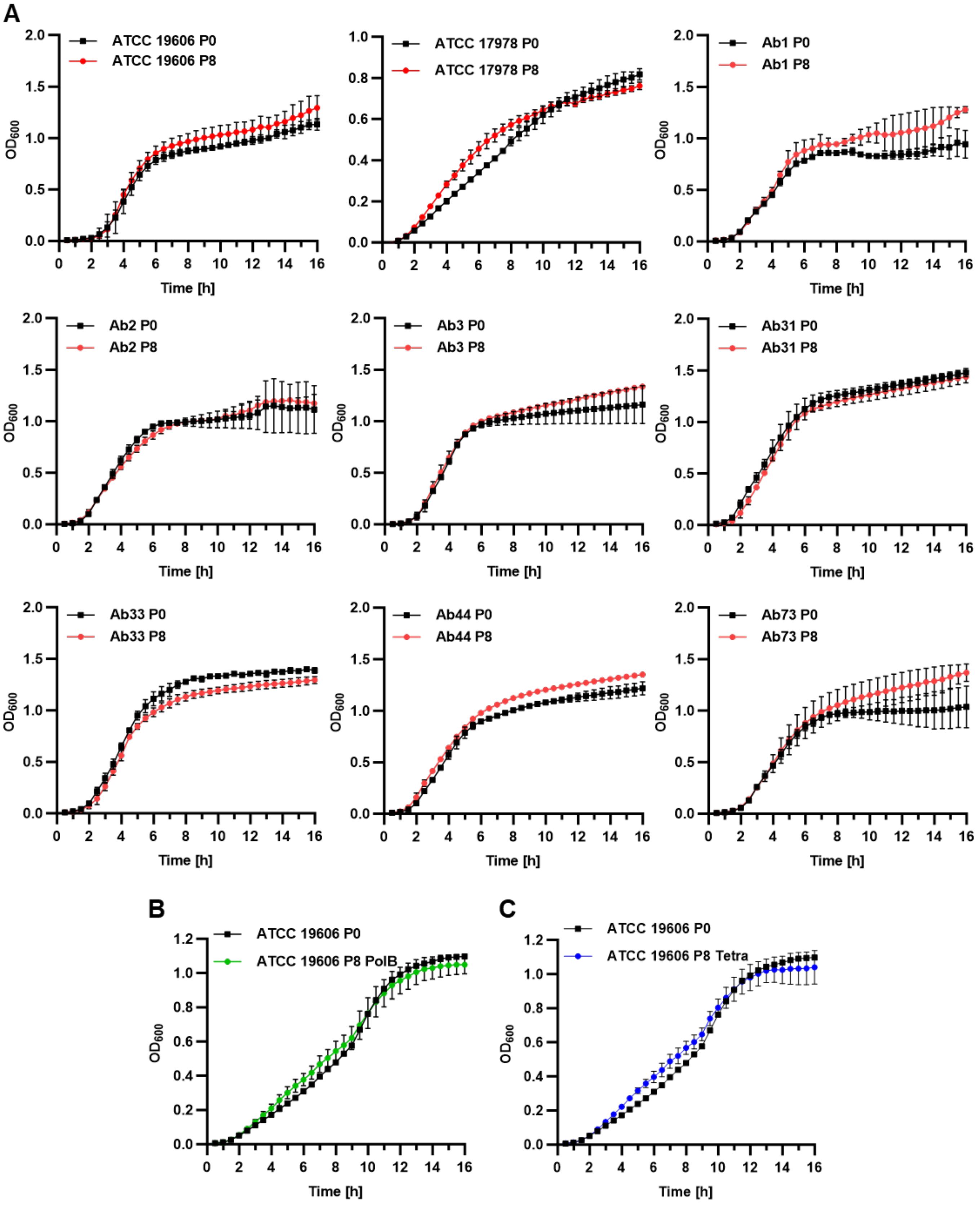

**Figure S2. Resistance acquisition does not induce in vitro growth defects in presence of increased salt concentration.** P0 and P8 isolates upon selection with TAT-RasGAP$_{317\text{-}326}$ **(A)**, polymyxin B **(B)** or tetracycline **(C)** were grown overnight in LB and diluted to an $OD_{600}$ of 0.01 in fresh LB containing 2% of NaCl. Growth was then assessed by $OD_{600}$ measurement each 30 minutes for a total of 16 hours. Values are the average of three experiments. Error bars represent standard deviation of the three replicates.

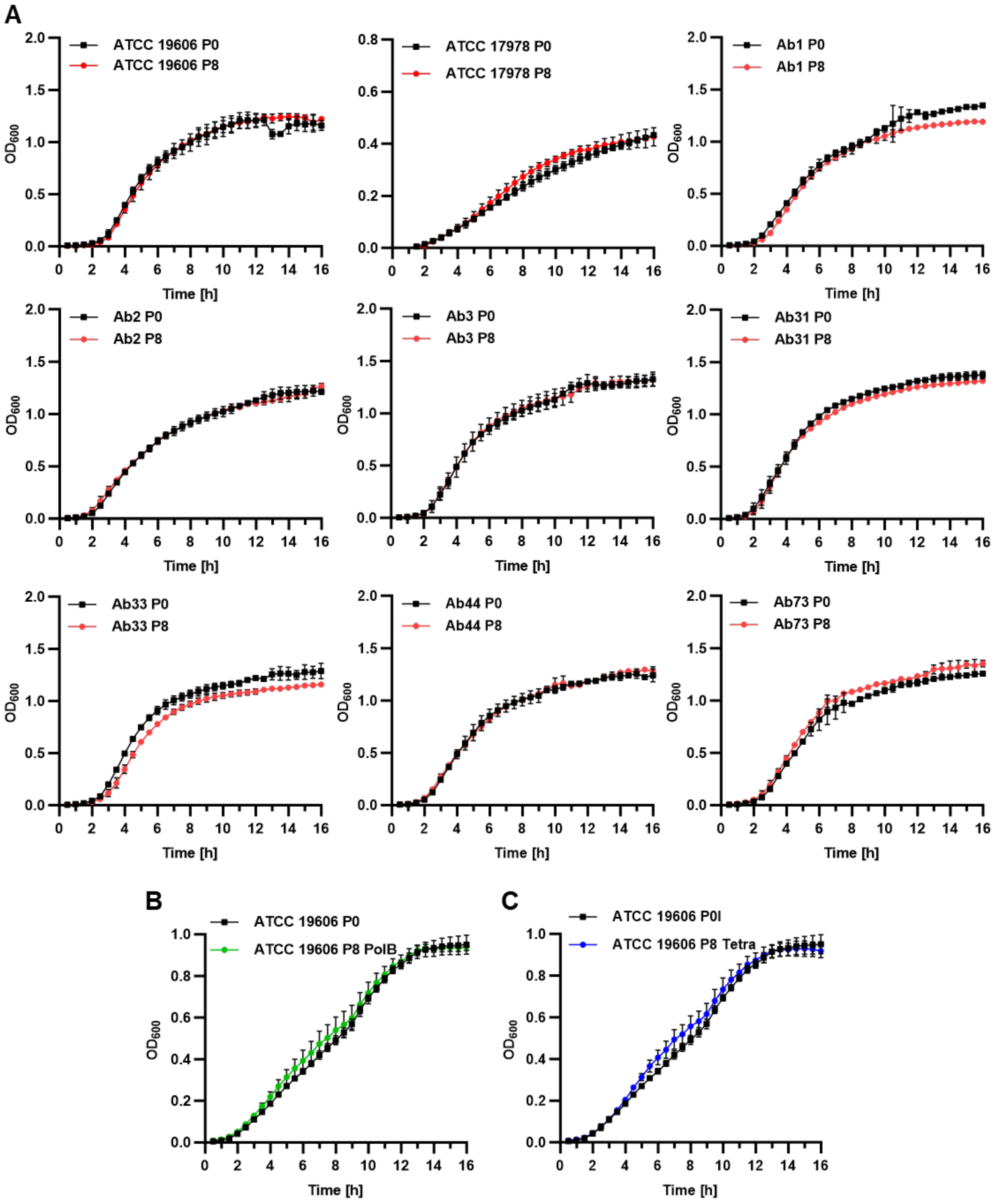

**Figure S3. Resistance acquisition does not induce morphology in vitro.** P0 and P8 isolates upon selection with TAT-RasGAP$_{317-326}$, polymyxin B or tetracycline were grown overnight in LB, diluted to an $OD_{600}$ of 0.1 in fresh medium and grown for 2 hours. Morphology of the different isolates was then assessed by microscopy.

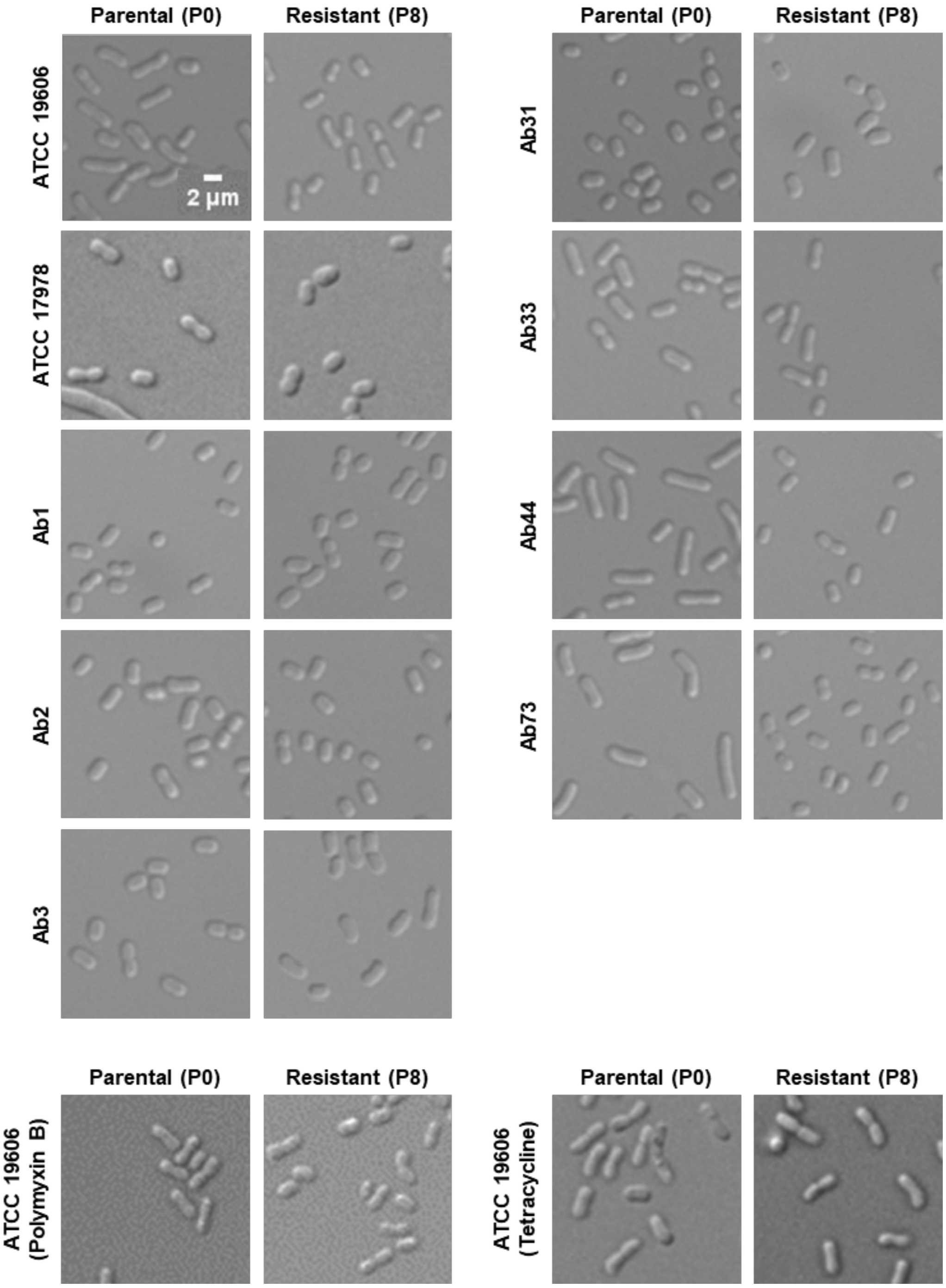

**Figure S4.** The effect of resistance acquisition on the structure of the biofilm was assessed by live-dead staining followed by Z-stacks acquisition with a confocal microscope. Reconstruction of Z projections are shown on the top and on the right for each strain. This figure is a complement to Figure 3.

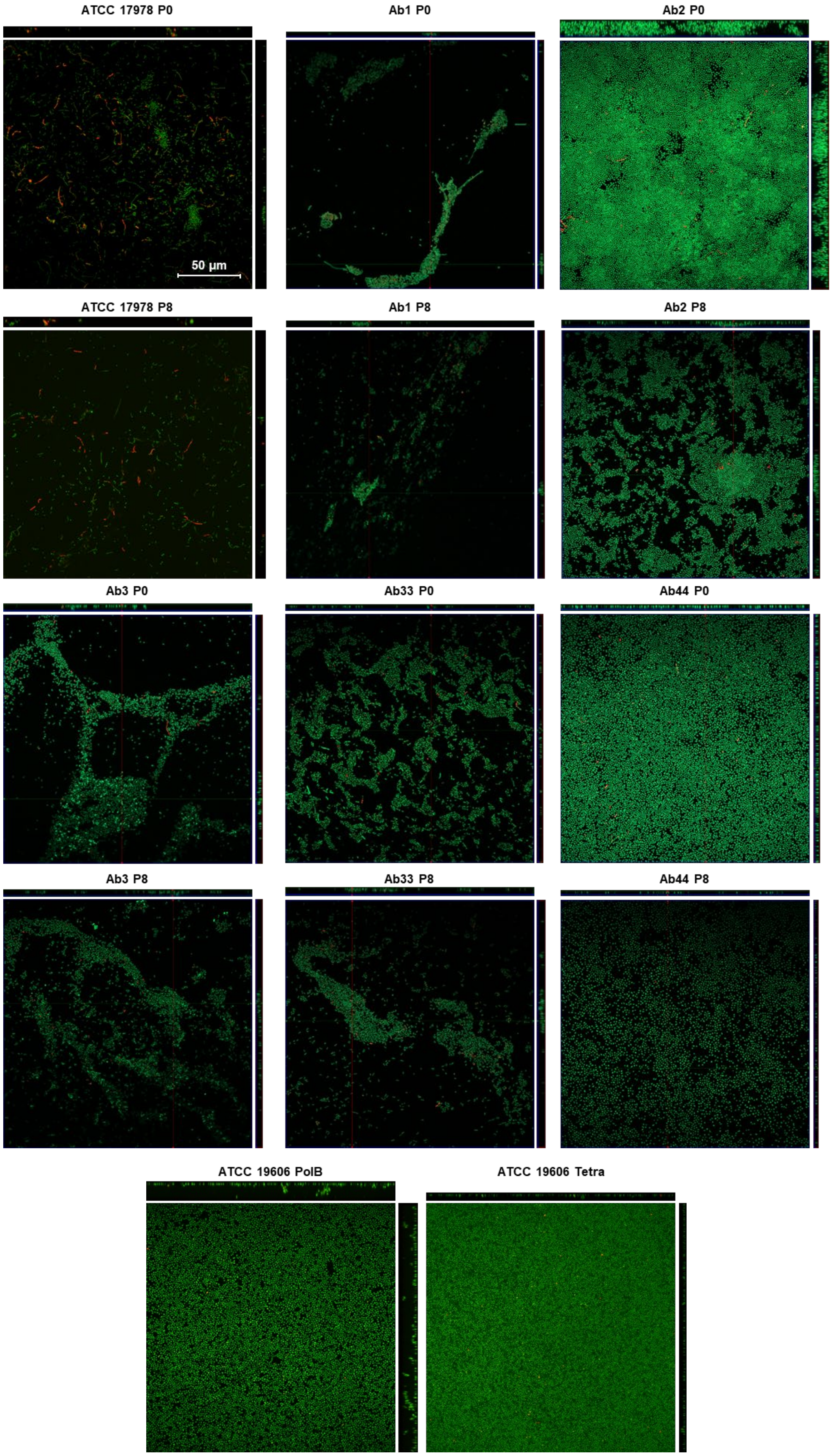

**Figure S5. MIC of TAT-RasGAP$_{317\text{-}326}$ (Black) and polymyxin B (Red) of all resistant strains selected in this study.**
MICs were measured on the indicated strains in duplicates. Black lines represent the mean of the replicates.

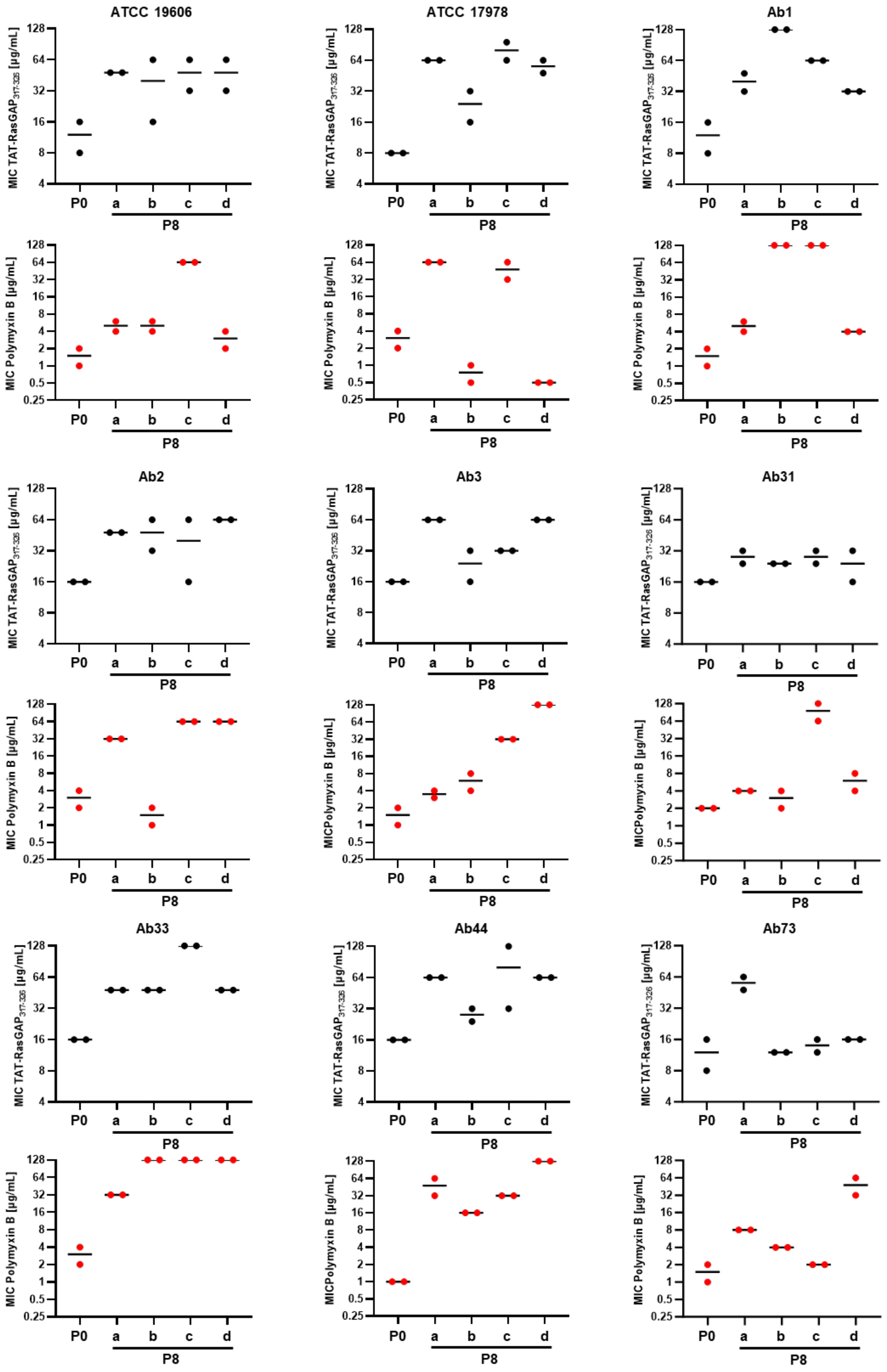

**Figure S6. Cross-resistance to polymyxin B is stable upon 8 passages in absence of drug selection.** ATCC19606 parental strain and selected resistant isolates b, c and d were passaged in absence of any antimicrobial agents for a total of 8 passages. MICs of TAT-RasGAP$_{317-326}$ and polymyxin B before (Resistant) and after (Back-selection) the 8 passages were measured.

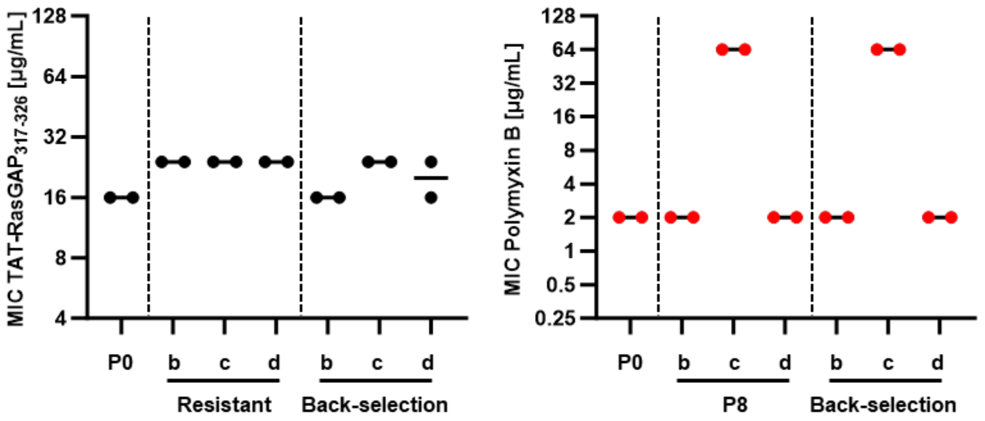

**Table S1:** Mutations detected in resistant strains, selected with TAT-RasGAP$_{317-326}$ (TAT), polymyxin B (PolB) or tetracycline (Tetra) from Tables 2 and 3. Type of mutations, as well as the targeted gene and the amino acid changes induced are shown. Ref nt: reference nucleotide, Mut nt: mutated nucleotide, Aa: amino acid.

| Strain | Contig n# | Position | Ref nt | Mut nt | Mutation type | Gene name | Aa change | Gene ID | Predicted gene product | Protein_ID | Aa length |
|---|---|---|---|---|---|---|---|---|---|---|---|
| 19606_TAT | 33 | 19751 | C | A | Missense | *ompA* | A63S | bakta_11390 | Outer membrane protein | Q6RYW5.1 | 356 |
| 19606_PolB | 7 | 37849 | G | T | missense | *mlaF* | Q96H | bakta_03235 | Toluene tolerance protein | QXV68106.1 | 272 |
| 19606_PolB | 34 | 41531 | A | T | missense | *pmrB* | M145K | bakta_11690 | Sensor histidine kinase | QXV68398.1 | 444 |
| 19606_Tetra | 7 | 60285 | G | C | missense | *rpsJ* | V57L | bakta_03345 | 30S ribosomal protein S10 | QXV68128.1 | 103 |
| 19606_Tetra | 33 | 37873 | C | A | stop_gained | | E139* | bakta_11470 | Methyltransferase | QXV68296.1 | 305 |
| 17978_TAT | 1 | 244138 | C | T | missense | *pmrA* | A14V | CHUV_01160 | DNA-binding response regulator | QFQ06527.1 | 224 |
| 17978_TAT | 1 | 244155 | T | G | missense | *pmrA* | L20V | CHUV_01160 | DNA-binding response regulator | QFQ06527.1 | 224 |
| Ab1_TAT | 3 | 18949 | T | A | missense | *tauE* | E71V | CHUV_00935 | Sulfite exporter | QXV68055.1 | 258 |
| Ab1_TAT | 5 | 27308 | A | C | missense | | T260P | CHUV_01620 | Hypothetical protein | QXV70740.1 | 333 |
| Ab1_TAT | 7 | 1245 | TTCAC | T | frameshift | *uhpC* | S67fs | CHUV_02195 | nitrate transmembrane transporter | QXV69291.1 | 449 |
| Ab1_TAT | 10 | 21166 | T | C | missense | *topA* | V791A | CHUV_03150 | DNA topoisomerase | QXV70887.1 | 878 |
| Ab1_TAT | 20 | 36632 | G | T | missense | *yaaA* | T142N | CHUV_05580 | peroxide stress protein | QXV68890.1 | 257 |
| Ab1_TAT | 45 | 14463 | C | T | missense | *bauA* | A391T | CHUV_09155 | TonB-dependent siderophore receptor | QXV68708.1 | 757 |
| Ab1_TAT | 51 | 3253 | C | T | missense | | G64E | CHUV_09845 | DUF2237 domain-containing protein | QXV69640.1 | 127 |
| Ab1_TAT | 63 | 5267 | G | A | missense | *mdh* | T184I | CHUV_11155 | malate dehydrogenase | QXV68186.1 | 328 |
| Ab1_TAT | 110 | 5006 | T | G | missense | | D185E | CHUV_14570 | phage capsid | No homologue | 368 |
| Ab1_TAT | 124 | 1494 | T | G | missense | | V458G | CHUV_15175 | Trimeric autotransporter adhesin | QXV70340.1 | 2258 |
| Ab1_TAT | 141 | 2607 | CGTGATG | C | inframe_deletion | *czcD* | H167_H168del | CHUV_15935 | Co/Zn/Cd efflux system component | QXV71353.1 | 329 |
| Ab1_TAT | 166 | 4646 | C | CA | frameshift | | D369fs | CHUV_16785 | PucR family transcriptional regulator | No homologue | 1443 |
| Ab1_TAT | 191 | 2689 | C | A | missense | | M132I | CHUV_17385 | Hypothetical protein | QXV70197.1 | 358 |
| Ab1_TAT | 191 | 2696 | T | C | missense | | E130G | CHUV_17385 | Hypothetical protein | QXV70197.1 | 358 |
| Ab1_TAT | 191 | 2718 | C | T | missense | | V123I | CHUV_17385 | Hypothetical protein | QXV70197.1 | 358 |
| Ab1_TAT | 242 | 434 | CCGGTCGGCTTGAG | C | frameshift | | A205fs | CHUV_18135 | GNAT family N-acetyltransferase | No homologue | 220 |
| Ab1_TAT | 261 | 261 | C | CA | frameshift | *proC* | L78fs | CHUV_18360 | pyrroline-5-carboxylate reductase | QXV70080.1 | 271 |
| Ab1_TAT | 279 | 198 | C | T | missense | | A301V | CHUV_18495 | lipid A phosphoethanolamine transferase | QXV68396.1 | 533 |
| Ab1_TAT | 291 | 280 | T | C | missense | | V86A | CHUV_18570 | tape measure protein (phage) | QXV68840.1 | 1637 |
| Ab1_TAT | 291 | 396 | C | T | missense | | L125F | CHUV_18570 | tape measure protein (phage) | QXV68840.1 | 1637 |
| Ab2_TAT | 3 | 22478 | A | C | missense | *pmrB* | T187P | CHUV_00950 | Sensor histidine kinase | QXV68398.1 | 444 |
| Ab2_TAT | 399 | 26 | C | T | missense | | M112I | CHUV_19810 | DUF4265 domain-containing protein | QXV70250.1 | 148 |
| Ab3_TAT | 58 | 22402 | C | A | missense | | V14L | CHUV_13180 | AAA family ATPase | No homologue | 552 |
| Ab3_TAT | 58 | 22414 | C | A | missense | | V10L | CHUV_13180 | AAA family ATPase | No homologue | 552 |
| Ab3_TAT | 156 | 64 | G | T | missense | | S286Y | CHUV_18030 | Integrase family protein | QXV69689.1 | 410 |
| Ab33_TAT | 61 | 18919 | G | A | missense | *pmrB* | P233S | CHUV_13230 | Sensor histidine kinase | QXV68398.1 | 444 |
| Ab44_TAT | 53 | 7269 | G | GA | frameshift | *acyC* | M452fs | CHUV_10515 | Adenylate/Guanylate cyclase | QXV69577.1 | 489 |
| Ab44_TAT | 133 | 6748 | T | A | missense | *pmrB* | I164F | CHUV_16740 | Sensor histidine kinase | QXV68398.1 | 444 |
| Ab73_TAT | 23 | 26937 | AT | A | frameshift | *pldA* | I80fs | bakta_04850 | Phospholipase | QXV69240.1 | 383 |
| Ab73_TAT | 32 | 10879 | A | C | missense | *bamA* | S693R | bakta_06200 | Outer membrane assembly protein | QXV69181.1 | 835 |
| Ab73_TAT | 35 | 24475 | A | T | missense | *lptC* | V146E | bakta_06675 | LPS export ABC transporter periplasmic protein | QXV70013.1 | 182 |
| Ab73_TAT | 101 | 4454 | T | A | stop_gained | *plcD* | L266* | bakta_13280 | Phospholipase | QXV68216.1 | 541 |

**Table S2:** Mutations detected in cross-resistant strains that do not have mutations in *pmrAB* (Ab2c, Ab3c) from Table 4. Type of mutations, as well as the targeted gene and the amino acid changes induced are shown. Ref nt: reference nucleotide, Mut nt: mutated nucleotide, Aa: amino acid.

| Strain | Contig n# | Position | Ref nt | Mut nt | Mutation type | Gene name | Aa change | Gene ID | Predicted gene product | Protein_ID | Aa length |
|---|---|---|---|---|---|---|---|---|---|---|---|
| Ab2c_TAT | 150 | 6550 | C | T | start_lost | *crp* | M1? | CHUV_14980 | Cyclic AMP receptor protein | QXV70134.1 | 235 |
| Ab2c_TAT | 223 | 6 | T | G | missense | *basI* | I230L | CHUV_17560 | acinetobactin biosynthesis phosphopantetheinyl transferase | QXV68718.1 | 251 |
| Ab2c_TAT | 223 | 21 | C | A | missense | *basI* | V225L | CHUV_17560 | acinetobactin biosynthesis phosphopantetheinyl transferase | QXV68718.1 | 251 |
| Ab2c_TAT | 223 | 146 | C | A | missense | *basI* | C183F | CHUV_17560 | acinetobactin biosynthesis phosphopantetheinyl transferase | QXV68718.1 | 251 |
| Ab2c_TAT | 243 | 3877 | A | T | missense | | F96I | CHUV_18000 | hypothetical protein | QXV70077.1 | 114 |
| Ab2c_TAT | 284 | 28 | T | A | missense | | T289S | CHUV_18690 | Hydrolase-4 domain-containing protein | No homologue | 293 |
| Ab2c_TAT | 291 | 145 | C | A | missense | | D101Y | CHUV_18790 | hypothetical protein | QXV70461.1 | 144 |
| Ab2c_TAT | 292 | 85 | T | A | missense | *uvrA* | S2T | CHUV_18810 | thiamine ABC transporter permease | QXV69420.1 | 753 |
| Ab2c_TAT | 303 | 1732 | G | T | missense | | A503E | CHUV_18960 | hypothetical protein | No homologue | 557 |
| Ab2c_TAT | 399 | 26 | C | T | missense | | M112I | CHUV_19810 | DUF4265 domain-containing protein | QXV70250.1 | 148 |
| Ab3c_TAT | 58 | 22402 | C | A | missense | | V14L | CHUV_13180 | AAA family ATPase | No homologue | 552 |
| Ab3c_TAT | 58 | 22414 | C | A | missense | | V10L | CHUV_13180 | AAA family ATPase | No homologue | 552 |
| Ab3c_TAT | 156 | 64 | G | T | missense | | S286Y | CHUV_18030 | Integrase family protein | QXV69689.1 | 410 |

**Table S3:** List of bacterial strains and oligonucleotides used in this study.

| Bacterial species | Bacterial strain | Source | Internal reference |
|---|---|---|---|
| *A. baumannii* | ATCC 19606 | ATCC | NJ629 |
| *A. baumannii* | ATCC 17978 | ATCC | NJ836 |
| *A. baumannii* | Ab1 | Heulot et al., 2017 | NJ721 |
| *A. baumannii* | Ab2 | Heulot et al., 2017 | NJ722 |
| *A. baumannii* | Ab3 | Heulot et al., 2017 | NJ723 |
| *A. baumannii* | Ab31 | Leshkasheli et al., 2019 | NJ749 |
| *A. baumannii* | Ab33 | Leshkasheli et al., 2019 | NJ750 |
| *A. baumannii* | Ab44 | Leshkasheli et al., 2019 | NJ751 |
| *A. baumannii* | Ab73 | Leshkasheli et al., 2019 | NJ752 |
| *E. coli* | DH5α pCasAb-apr | Wang et al., 2019 | NJ823 |
| *E. coli* | DH5α pSGAb-km | Wang et al., 2019 | NJ824 |
| *E. coli* | DH5α pSGAb-spe | Wang et al., 2019 | NJ825 |

| Oligonucleotides | Sequence | Purpose | Origin |
|---|---|---|---|
| PmrB_450_F | AAT TAT TTT ACC TTT TGC A | *pmrB* amplification and sequencing | This study |
| PmrB_950_R | ACA AAA CCT AAA TCG ATT T | *pmrB* amplification and sequencing | This study |
| PmrB_50_F | GTG TCA TCT TAG GTT GTA TTT | *pmrB* amplification and sequencing | This study |
| PmrB_1260_R | CTT ATC GAG AGT TAA AGT CC | *pmrB* amplification and sequencing | This study |
| PmrB_spacerF_17978 | TAG TTT CAA TTG GTG TGA GTT CTT | targeted mutagenesis of *pmrB* | This study |
| PmrB_spacerR_17978 | AAA CAA GAA CTC ACA CCA ATT GAA | targeted mutagenesis of *pmrB* | This study |
| PmrB_Donor | AGG TAA AAG CTC TTG AGG ATA ATC ATG TAC TTC AATTGG GGG GAG CTC TTC GGA ATC GCG TTC TTT TAA CTCATT TTT AA | targeted mutagenesis of *pmrB* | This study |
| pCasAb_5053F | AGT CTA ATA GAA TGA GGT CG | targeted mutagenesis of *pmrB* | Wang et al., 2019 |
| pCasAB_5647R | TGA TAG AAC ATG TAA ATC GA | targeted mutagenesis of *pmrB* | Wang et al., 2019 |
| M13R | CAG GAA ACA GCT ATG ACC | targeted mutagenesis of *pmrB* | Wang et al., 2019 |
